# Pathophysiology and clinical consequences of arterial blood gases and pH after cardiac arrest

**DOI:** 10.1186/s40635-020-00307-1

**Published:** 2020-12-18

**Authors:** Chiara Robba, Dorota Siwicka-Gieroba, Andras Sikter, Denise Battaglini, Wojciech Dąbrowski, Marcus J. Schultz, Evert de Jonge, Chloe Grim, Patricia RM Rocco, Paolo Pelosi

**Affiliations:** 1 Anesthesia and Intensive Care, San Martino Policlinico Hospital, IRCCS for Oncology and Neurosciences, University of Genoa, Largo Rosanna Benzi, 15, 16100 Genoa, Italy; 2 Department of Anaesthesiology and Intensive Therapy, Medical University of Lublin, Lublin, Poland; 3 Internal Medicine, Municipal Clinic of Szentendre, Szentendre, Hungary; 4 Department of Intensive Care, Amsterdam University Medical Centers, location ‘AMC’, Amsterdam, The Netherlands; 5 Department of Intensive Care, Leiden University Medical Center, Leiden, The Netherlands; 6 Laboratory of Pulmonary Investigation, Carlos Chagas Filho Institute of Biophysics, Federal University of Rio de Janeiro, Rio de Janeiro, Brazil; 7 Department of Surgical Sciences and Integrated Diagnostics, University of Genoa, Genoa, Italy

**Keywords:** Cardiac arrest, Ventilator targets, Intracellular acidosis, Catecholamine, Gas exchanges

## Abstract

Post cardiac arrest syndrome is associated with high morbidity and mortality, which is related not only to a poor neurological outcome but also to respiratory and cardiovascular dysfunctions. The control of gas exchange, and in particular oxygenation and carbon dioxide levels, is fundamental in mechanically ventilated patients after resuscitation, as arterial blood gases derangement might have important effects on the cerebral blood flow and systemic physiology.

In particular, the pathophysiological role of carbon dioxide (CO_2_) levels is strongly underestimated, as its alterations quickly affect also the changes of intracellular pH, and consequently influence metabolic energy and oxygen demand. Hypo/hypercapnia, as well as mechanical ventilation during and after resuscitation, can affect CO_2_ levels and trigger a dangerous pathophysiological vicious circle related to the relationship between pH, cellular demand, and catecholamine levels. The developing hypocapnia can nullify the beneficial effects of the hypothermia. The aim of this review was to describe the pathophysiology and clinical consequences of arterial blood gases and pH after cardiac arrest.

According to our findings, the optimal ventilator strategies in post cardiac arrest patients are not fully understood, and oxygen and carbon dioxide targets should take in consideration a complex pattern of pathophysiological factors. Further studies are warranted to define the optimal settings of mechanical ventilation in patients after cardiac arrest.

## Background

The aim of cardiopulmonary resuscitation is to promptly restore spontaneous circulation in order to avoid hypoxic ischemic brain injury. In this context, ventilation has the goal to maintain an appropriate level of arterial oxygen (PaO_2_), while reducing arterial carbon dioxide levels (PaCO_2_), avoiding respiratory acidosis. However, the pathophysiology of cardiac arrest and its systemic effects, as well as the relationship between arterial blood gases, hemodynamic management, and ventilation during and after resuscitation is complex. Evidence regarding the optimal oxygen or carbon dioxide (CO_2_) targets is limited, and the relationship between PaO_2_ and PaCO_2_ targets in the context of catecholamine administration and pH changes is not fully understood. Also, the pathophysiological effects of CO_2_ are underestimated in the literature; variations of carbon dioxide levels can quickly change the extracellular-intracellular concentration of H^+^, and modifications in pH and acidosis have extraordinary importance on systemic homeostasis [1].

The aim of this review is to discuss the pathophysiology of arterial blood gases and pH in mechanically ventilated patients after cardiac arrest and their clinical consequences. We will not focus on ventilator settings but on the pH, PaO_2_, and CO_2_ physiological targets to achieve in this cohort of patients.

## Pathophysiological basis

### Acidosis and lactate

**Fig. 1:**
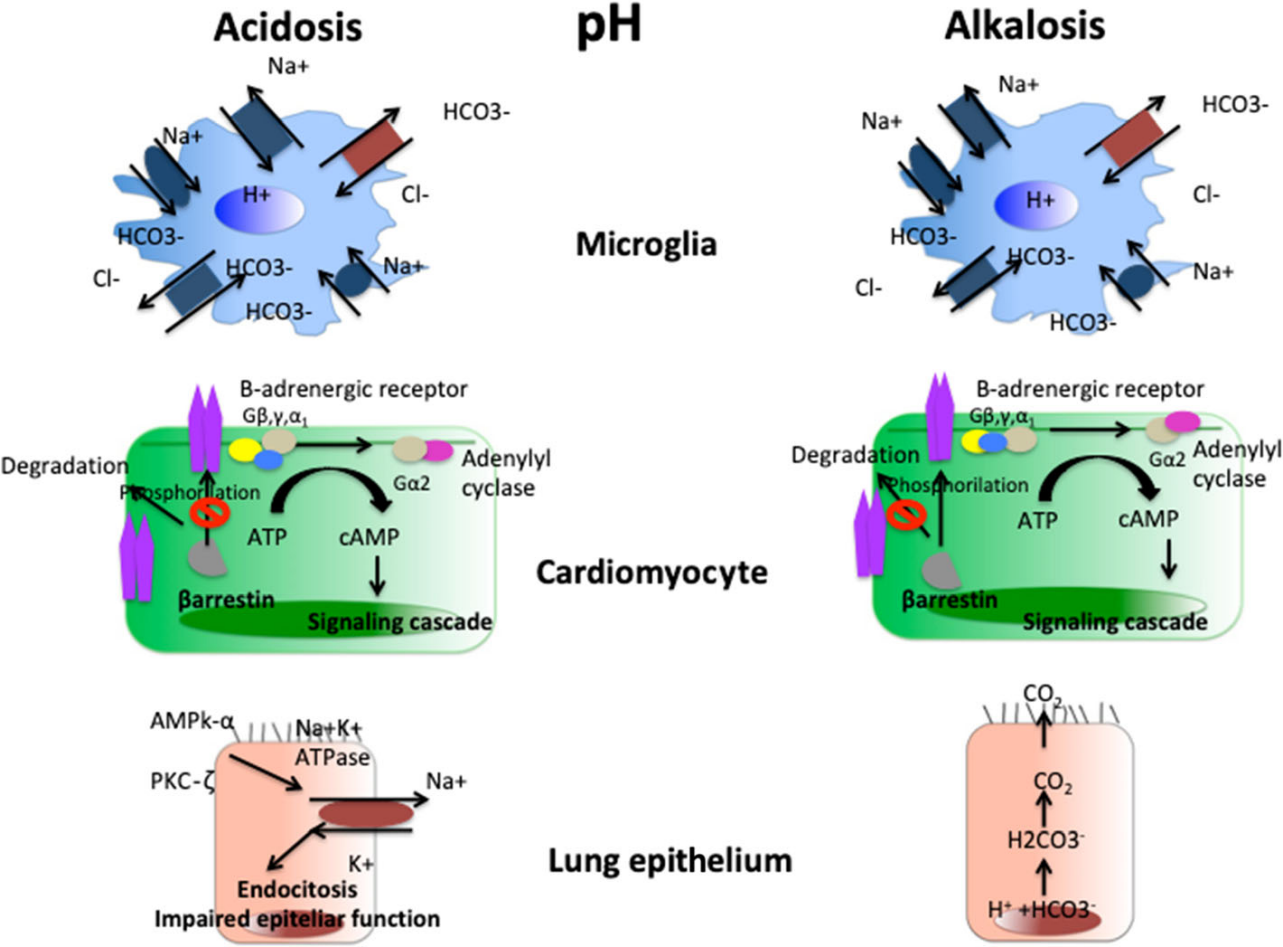
Biological effects of pH on microglia, cardiomyocytes, and lung epithelial cells. Microglia expresses two classes of acid-base transporting proteins: acid loaders (red) and acid extruders (blue). In acidosis, acid extruder proteins are increased while in alkalosis these are downregulated. In cardiomyocytes, the regulation of the adrenergic receptors is determined by β-arrestins, which can trigger the internalization process of dephosphorization or degradation and therefore define the up- and downregulation of the internalized molecule either to recycling or degradation, respectively. In acidosis, adrenergic receptors are downregulated and less responsive to catecholamines. In the lung epithelium, reduction of lung edema clearance is associated with the endocytosis of the Na+/K+-ATPase from the plasma membrane of alveolar epithelial cells, which leads to decreased Na+/K+-ATPase activity. During acidosis, protein kinase C (PKC)-ζ phosphorylates the Na+/K+-ATPase α1-subunit, leading to endocytosis of the Na+/K+-ATPase. The activation of PKC-ζ is regulated by AMP kinase (AMPK). Acidosis culminates in the Na+/K+-ATPase endocytosis from the cell plasma membrane. In the lung epithelium, alkalosis with low carbon dioxide and hyperventilation can determine increase of mechanical power and activation of the inflammatory system

**Fig. 2:**
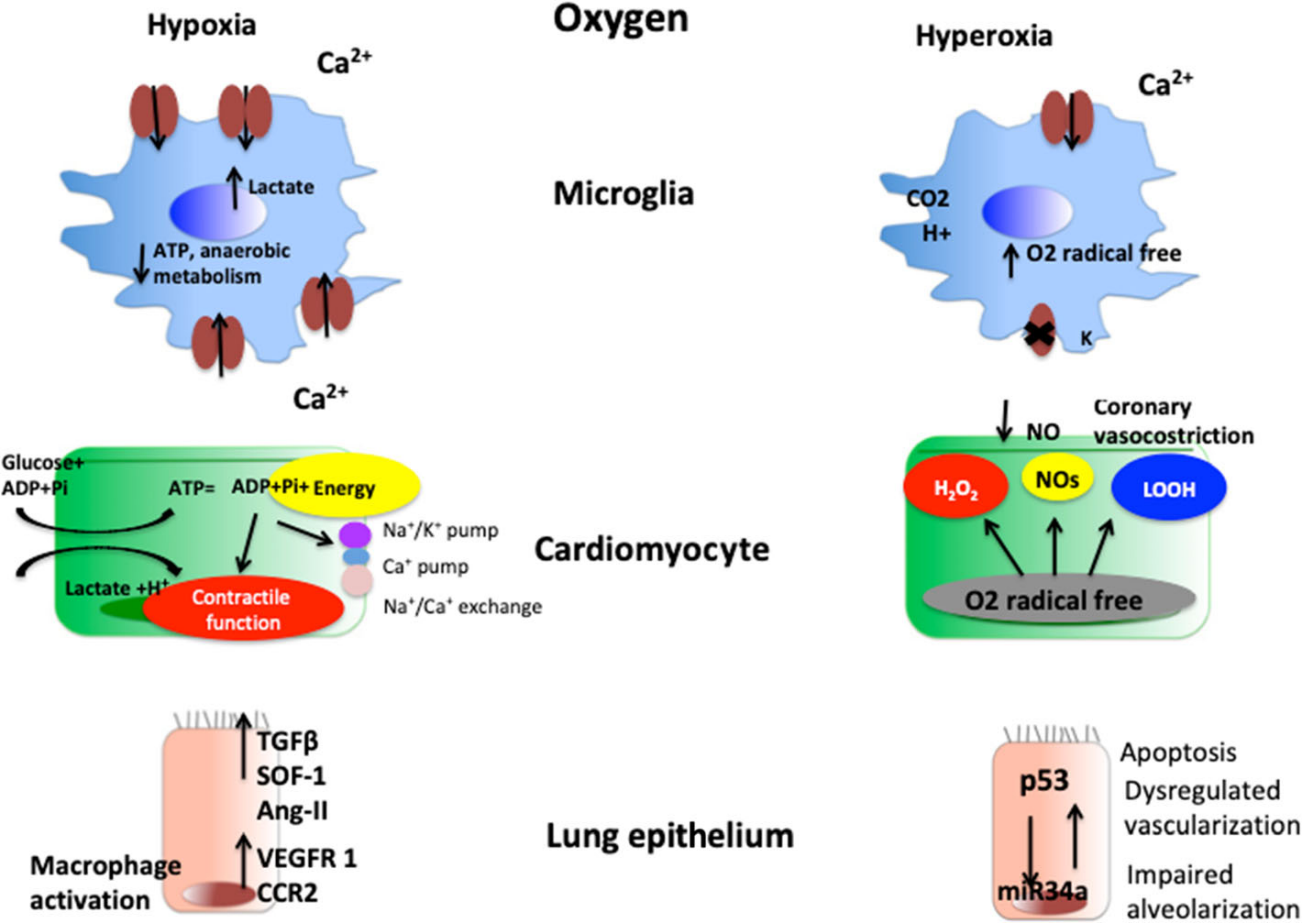
Effect of oxygen on microglia, cardiomyocytes, and lung epithelial cells. Microglia: hypoxic neurons have an anaerobic metabolism with increased intracellular Ca^2+^ intake; persistent hypoxia generates reduction of ATP and further energetic failure. Hyperoxia can disrupt microglia function by increasing free radicals. Hyperoxia increases input resistance to antioxidant and decrease membrane conductance (K+ channel) and stimulates firing of putative central CO_2_/H^+^ chemoreceptors neurons. Cardiomyocytes: effects of anoxia on cellular energetic turnover and on intra- and extracellular environments and its effect on cardiac function. In hyperoxia, O_2_ radical free and reduction of nitric oxide can eventually lead to coronary vasoconstriction. In the lung epithelium, hypoxia induces pulmonary vascular remodeling, with resident vascular cell activation, monocytes/fibrocytes recruitment, and persistent vasoconstriction structural remodeling. Hyperoxia exposure stimulates p53 to activate miR34a in a positive feedback loop, with consequent abnormal cell proliferation, apoptosis, impaired alveolarization, and cell death

**Fig. 3:**
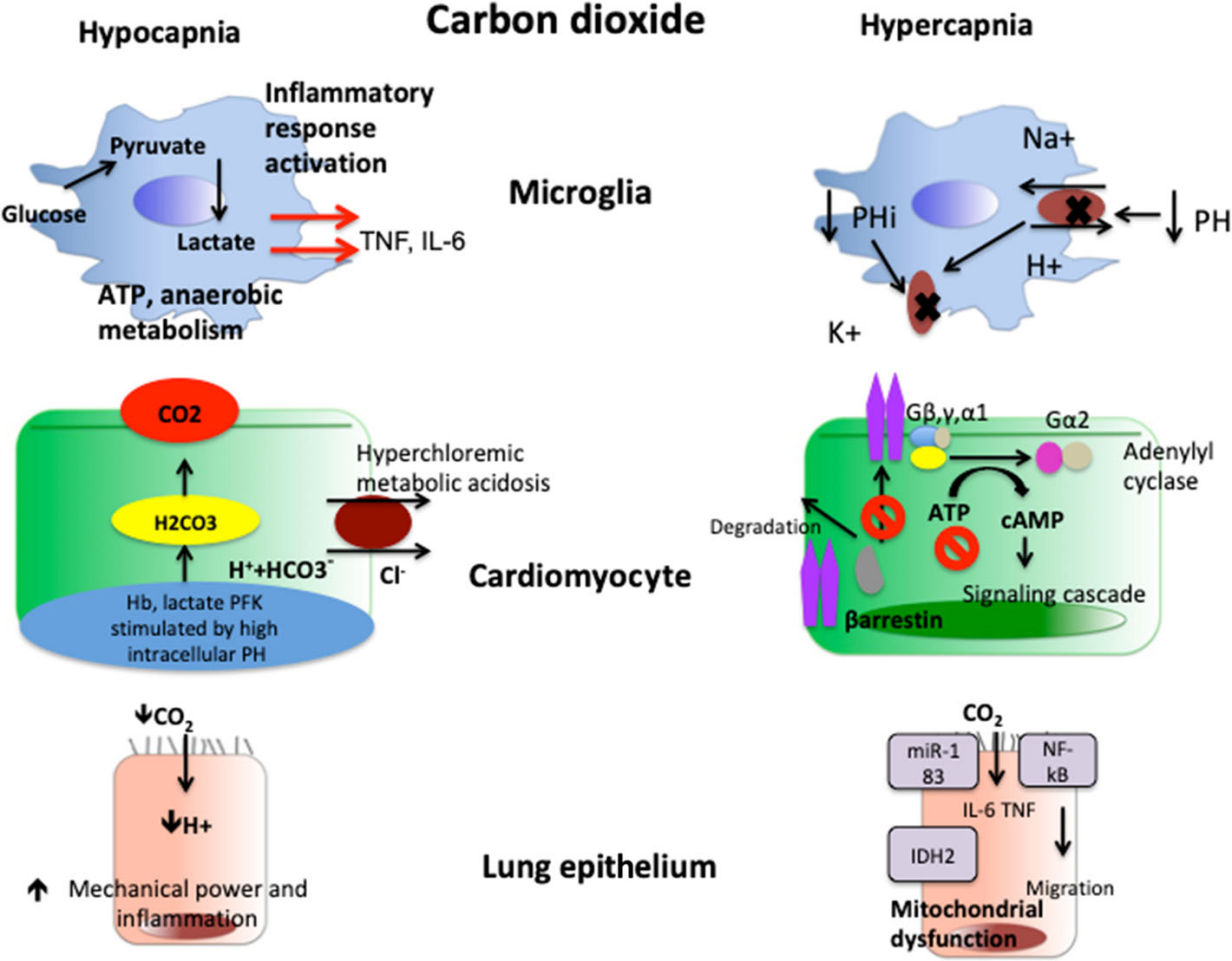
Effect of carbon dioxide on microglia, cardiomyocytes, and lung epithelial cells. Microglia: hypercapnia decreases extracellular pH and intracellular pH (Phi). Increased Phi increases firing rate of CO_2_/H_2_ chemosensitive neurons, by an oxidant-induced decrease in K+ conductance. Hypocapnia with cerebral vasoconstriction and ischemic insult shifts the anaerobic metabolism and activates local and systemic inflammatory response. Cardiomyocytes: hypercapnia and acidosis reduce the sensitivity of the adrenergic receptor and expression. Hypocapnia has effect on intracellular buffers (mostly hemoglobin within red blood cells) determining release of hydrogen. Hydrogen combines with bicarbonate to form carbonic acid, which then disassociates to form water and CO_2_, thus replenishing the depleted PaCO_2_. Lung epithelium: hypercapnia inhibits proliferation of alveolar epithelial cells due to mitochondrial dysfunction resulting from hypercapnia-induced miR-183 which downregulates the TCA cycle enzyme isocitrate dehydrogenase-2 (IDH). Hypercapnic acidosis impairs alveolar epithelial cell migration by the NF-kB dependent mechanism. Hypercapnia inhibits mRNA and protein expression of IL-6 and TNF and decreases phagocytosis in macrophages. Hypocapnia and hyperventilation can determine increase of mechanical power and activation of the inflammatory system. CO_2_, carbon dioxide; PaCO_2_, partial pressure of carbon dioxide; TCA, tricarboxylic acid cycle; NF-kB, nuclear factor kappa-light-chain-enhancer of activated B cells; IDH, enzyme isocitrate dehydrogenase-2; TNF, tumor necrosis factor; IL, interleukin; mRNA, ribonucleic acid

Acute circulatory shock and cardiac arrest predispose to inadequate oxygen delivery, resulting in cellular hypoxia and acute brain damage [2] (Figs. 1, 2, 3).

Patients after cardiac arrest may often present with a combination of metabolic and respiratory disturbances, where lactic acidosis is the main cause of metabolic acidosis (defined as pH ≤ 7.35 and lactatemia > 2.0 mmol l^−1^ with a PaCO_2_ ≤ 42 mmHg) [3, 4].

Hypoperfusion and acidotic disturbances during return of spontaneous circulation (ROSC) result in a misbalance between lactate production and removal; the dissociation of lactic acid produces hydrogen ions which are used for the oxidative phosphorylation and adenosine Tri-Phosphate (ATP) production [3, 5]. In a canine model, Sanders et al. [6] showed that metabolic acidosis is predominant in the late phase of cardiac arrest, while in the first short period after circulatory arrest, respiratory alkalosis is more frequent, probably related to artificial hyperventilation.

However, acid-base disturbances imply a larger spectrum of clinical consequences. Acute lactic acidosis depresses left ventricular contractility, triggers the release of endogenous catecholamine, and decreases the responsiveness of the left ventricle to the actions of catecholamine [7]. Therefore, late detection and treatment of acidosis in the context of impaired cardiovascular function and responsiveness to vasopressors in post cardiac arrest status may lead to worse outcome [8].

The pathophysiological effects of acidosis on cardiomyocytes are well described [2]; extracellular and intracellular acidosis reduces the number of adrenoreceptors expressed on the cell membrane, in particular beta receptors, and contributes to reduced contractile responsiveness to catecholamines [9]. Decreased pH also influences the synthesis and release of tumor necrosis factor (TNF-α) and nitric oxide (NO) in macrophages or macrophage-like cell lines and can “per se“ affect hemodynamic stability: moderate acidosis (pH = 7–7.20) might elevate nitric oxide (NO) production and levels, but severe acidosis (pH < 7.0) determinates a decrease in NO release and NO synthesis [10–13].

### Oxygen

After successful resuscitation and ROSC, achieving appropriate levels of oxygen and carbon dioxide in arterial blood is necessary to avoid further hypoxia and secondary damage [14] (Figs. 1, 2, 3). A large cohort study performed in 82 intensive care units (ICUs) in hospitals participating to the Dutch National Intensive Care Evaluation (NICE) [15] showed that both PaCO_2_ and PaO_2_ levels in post cardiac arrest patients are predictors for hospital mortality.

Re-oxygenation after the no flow phase is a major stimulant for myocardial catecholamine release [16]. Decreased arterial O_2_ levels are associated with low pH and almost immediate sodium-potassium (Na^+^/K^+^) ATPase dysfunction, with consequent ion imbalance. Thus, hypoxemia induces mitochondrial damage, which determinates systemic inflammation with energy failure and cell apoptosis [17]. On the other hand, even hyperoxia can be detrimental and has shown to be associated with higher mortality (EMShockNet investigators) [18].

Hyperoxia is usually observed after cardiac arrest as consequence of intubation and artificial ventilation with high fraction of oxygen (FiO_2_) [19]. Mild hyperoxia can be defined as arterial oxygen partial pressure higher than 120 mmHg (> 16 kPa) and severe hyperoxia as PaO_2_ > 200 mmHg (26.7 kPa). Elevated PaO_2_ may increase oxidative stress, releases reactive oxygen species (ROS), and predisposes to mitochondrial dysfunction that finally results in molecular injury including deoxyribonucleic acid (DNA) damage and worsened organ function.

Elevated ribonucleic acid (RNA) expression of nitric oxide synthase (iNOS) was observed during hyperoxia when compared to normoxia in animal models of ischemia/reperfusion injury. Furthermore, pro apoptotic factors such as cyclooxygenase-2 (COX-2), toll like receptor 4 (TLR-4), and caspase-3 protein expression were increased, with a strong impact on apoptosis and inflammation mechanisms leading to myocardial injury [20].

Animal studies demonstrated improved outcome and lower neuronal damage using low O_2_ supplementation compared with high O_2_ supplementation. Recent data show an increased accumulation of hypoxic-induced factor (HIF) during exposure to oxygen FiO_2_ > 30% in the brain tissue, hepatocytes, and myocardium [21–23].

Translated in the clinical practice, hyperoxia toxicity, with overproduction of ROS and oxidative cellular injury, can determinate elevated levels of superoxide, inhibits hypoxic pulmonary vasoconstriction, affects the adsorption of atelectasis, and is cause of apoptosis, necrosis, and direct ROS toxicity (Lorrain Smith effect) on alveolar capillary barrier which predisposes to hemorrhagic pulmonary edema; also, hyperoxia leads to vasoconstriction of arteries to distant organ thus causing coronary spasm and ischemic events [24].

### Carbon dioxide

Youn-Jung Kim et al. [25] suggested that PaCO_2_ may be a marker of post ROSC cerebral ischemia in nontraumatic cardiac arrest as PaCO_2_ levels directly regulate cerebral blood flow and cerebrovascular diameter, with every 1 mmHg decrease of PaCO_2_ determining a decrease of approximately 3% in cerebral blood flow. Hypocarbia may cause cerebral vasoconstriction that worsens ischemic injury and should be avoided to preserve cerebral blood flow [26, 27]. On these basis, Eastwood et al. [28, 29] suggested that mild hypercapnia (PaCO_2_ 50–55 mmHg) may improve cerebral oxygenation when compared with normocarbia (PaCO_2_ 35–45 mmHg), and Beitler et al. [30] in a recent meta-analysis showed that neurocognitive outcome is improved by using low tidal volume ventilation. On the other hand, elevated levels of PaCO_2_ can have detrimental effects on patients’ outcome, especially if related to hypercapnic acidosis [31, 32].

### Catecholamines

Catecholamines are principally used in cardiac arrest to achieve hemodynamic stabilization and to improve systemic blood pressure and cardiac output in hemodynamically unstable patients. However, recent studies showed that epinephrine administration is associated with post-resuscitation myocardial dysfunction and worse outcome [33]. Synthetic and endogenous catecholamines predispose to direct organ damage and cause many immunological, metabolic, and coagulation disturbances [34]. Catecholamines have “two faces”—are necessary to survive, but they can harm when in excess [35]. Adverse effects of catecholamines are connected to β1 receptors binding, resulting in elevated myocardial oxygen consumption, myocardial failure, and left ventricular dysfunction. Raised levels of catecholamines predispose to β adrenoreceptors desensitization and downregulation of adrenoreceptors [36, 37]. Several conditions can increase plasma catecholamines up to toxic levels, including hypothyroidism, acidosis, and hypoxia as well as higher lactic acid [38].

Catecholamines can also affect peripheral vascular resistances through myosin light-chain kinase, which regulate smooth muscle cells myosin activity. The alpha 1 receptors activation induce vasoconstriction with increased risk of coronary spasms and can determinate reduction of splanchnic perfusion, lower mucosal pH, and higher hepatic vein lactate [39, 40]. Vasoconstriction, by increasing heart work, and ventricular overfilling predispose to ventricular arrhythmias.

Finally, catecholamine release, with consequent important alteration in microcirculatory system, impairs cardiac fatty-acid metabolism, and mitochondrial metabolism, and can activate immunology response. Neuronal and immune responses are rapidly activated during cardiac arrest and trigger a pathway of systemic neuroendocrine and immune responses with uncontrolled immune reaction because of inadequate hormonal suppression and disturbance between anti-inflammatory and proinflammatory responses. Catecholamines and their metabolites influence every aspect of innate and adaptive immune response, because immune cells express both alpha and beta adrenoreceptors [41]. In particular, catecholamines regulate T and B cells proliferation, differentiation, and apoptosis, and selectively activate lymphocytes subpopulations by release of pro- and anti-inflammatory mediators and are responsible for systemic activation and multiorgan damage [42].

## The pathophysiology of  the vicious circle after cardiac arrest

**Fig. 4:**
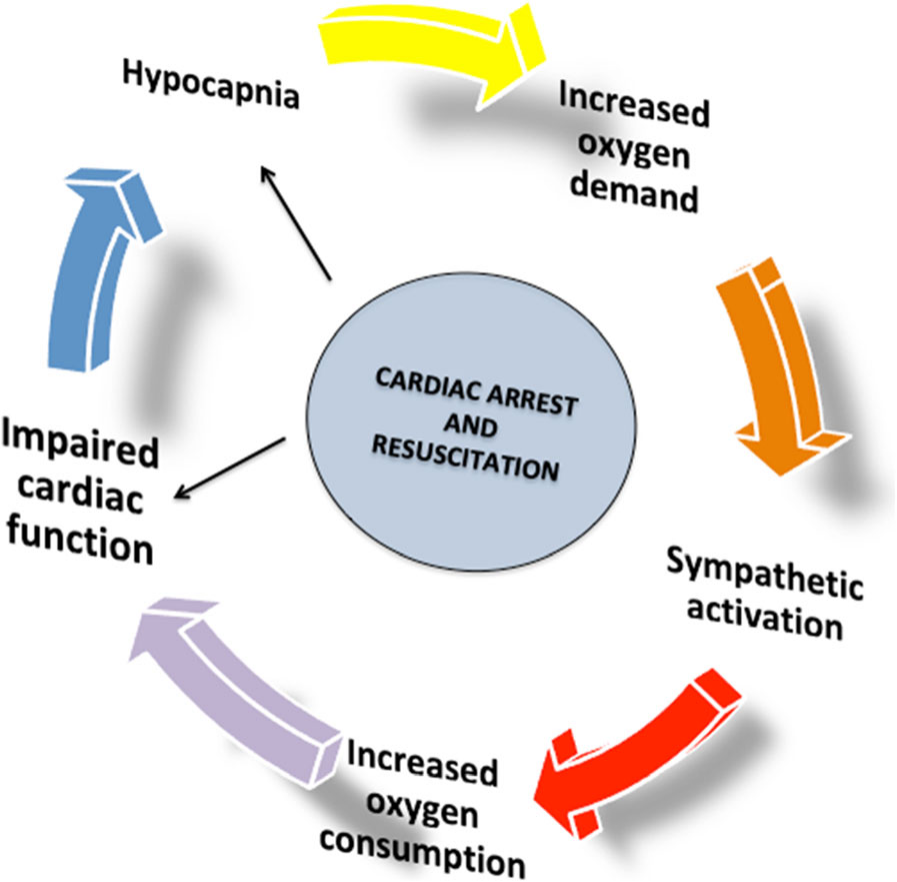
The pathophysiologic vicious circle after cardiac arrest: hyperventilation and hypocapnia can quickly cause intracellular and extracellular pH increase; this increases metabolic energy and O_2_ demand, while ATP production is reduced in cardiac arrest for the activation of the anaerobic pathway; this determinates a sympathetic-like activation aimed to compensate the increased energy demand. Also, cardiac arrest per se' and pH derangements strongly influence intracellular function, thus increasing metabolic expenditure, and oxygen consumption. This occurs in the contest of cardiac injury related to the cardiac arrest as well as to pH changes which can further impair cardiac function; impaired myocardium function results in impaired diastolic function with further hyperventilation and hypocapnia. O_2_, oxygen; ATP, adenosine triphosphate

The above-described pathophysiological cross-talk between the immune system, catecholamine release, and metabolic status may result in the development of a dangerous pathophysiological vicious circle, where the ventilation and correction of arterial blood gases, as well as catecholamine administration may result in a paradox further progression of primary damage. In particular, the pathophysiologic effects of carbon dioxide have a strong impact on the context of catecholamine administration and pH variations, but these effects are still poorly understood. CO_2_ alterations during resuscitation can quickly cause abrupt intracellular and extracellular pH changes; hypocapnia, as consequence of hyperventilation, increases the demand of metabolic energy and O_2_, while ATP production is often reduced, thus determining a sympathetic-like activation which further increases the energy demand (Fig. 4).

Metabolic compensatory mechanisms consequent to acute hypocapnia or hypercapnia are very slow, e.g., the renal metabolic compensation takes 5–7 days to restore the original intracellular pH [43].

The pH derangements strongly influence the function of many enzymes as well as the active and passive cytoplasmic transport of ions, especially of calcium (Ca^2+^) [44, 45]. Therefore, intracellular alkalosis during acute hypocapnia leads to increased metabolic expenditure, oxygen consumption, and increased metabolic demand [46]. On the other hand, pH changes can further impair cardiac function, which is common after cardiac arrest; impaired myocardium function results in worsened relaxation ability and increased diastolic pressure, resulting in increased pulmonary wedge pressure (PWP) and further hyperventilation and hypocapnia according to the above-described mechanisms [47].

A vicious circles can therefore occur, triggered by hypocapnia, which determinates a lack of energy substrate together with cardiac and pulmonary failure [48] (Fig. 4).

The increased production of catecholamines is a compensatory phenomenon in acidosis, and there is a positive feedback between catecholamine production and H+ concentration: the catecholamine-sensitivity is very low in acidosis, though their production is high. However, when compensatory mechanisms and treatment improve acidosis and an alkalotic pH develops, the catecholamine levels remain still high; the synergism of the high levels of catecholamines and pH can poison systemic and immunological functions through the previously described mechanisms [47, 49].

Thus, the beneficial or harmful effect of catecholamines depends on different factors including pH, levels of PaCO_2_, and lactate. In particular, the production of lactate depends on three mechanisms: hypoxia/anoxia, with activation of anaerobic pathway (lactate/pyruvate); ATP demand and rate of metabolism (low in acidosis and high in intracellular alkalosis), which is pH dependent; and intracellular pH [50]. Also, when tissue hypoxia is resolved, lactate production will terminate, and lactate degradation will start; therefore, the rapidity and the magnitudo of the raise of carbon dioxide should ideally take into consideration the halftime of lactate degradation. Finally, lactate production is strictly determined by the pH of the intracellular space as it decreases in acidosis while it elevates in alkalosis.

Although from these pathophysiological concepts, it is clear that the best way to terminate this vicious circle is to elevate PaCO_2_ levels, as acidosis can slow down the enzymatic intracellular and membrane processes by reducing energy demand-we should avoid raising PaCO_2_ above normal level because this will lead to metabolic disorders in the long term [30].

## Clinical implications

Post cardiac arrest syndrome results in different mechanisms of injury, including ischemia/reperfusion injury, oxidative stress, microcirculatory disturbances with coagulopathy, and inflammation which may increase the risk for multiple organ failure (Table 1).

**Table 1:** Systemic consequences of cardiac arrest

	Pathophysiology	Clinical consequences
Ischemia -reperfusion injury	*√* Free radical formation *√* Apoptosis, necrosis *√* “Sterile inflammation” *√* Impaired resistance to infection *√* Endothelial damage *√* Impaired vasoregulation *√* Adrenal suppression *√* Electrolytes disturbances *√* Acidosis, hypoxia, catabolism	*√* Multiorgan failure *√* Infection/sepsis *√* Microbiome disturbances *√* Tissue hypoxia/ischemia *√* Hyperglycemia *√* Increase of intra-abdominal pressure *√* Development of abdominal compartment syndrome
Brain injury/global cerebral ischemia (GCI)	*√* Calcium homeostasis disruption *√* No reflow *√* Pyrexia *√* Hyperglycemia *√* Hyperoxygenation *√* Impaired cerebrovascular autoregulation *√* Impairment of hypothalamic-pituitary-adrenal axis *√* Raised corticotropin releasing factor (CRF)-blood flow changes	*√* Damage of blood-brain barrier (BBB) (endothelial dysfunction and capillary permeability) *√* Coma *√* Seizures *√* Cognitive dysfunction *√* Stroke *√* Brain death *√* Pulmonary complications following cerebral ischemia (neurogenic pulmonary edema, acute distress respiratory syndrome, pneumonia)
Myocardial dysfunction	*√* Global hypokinesis *√* Preserved coronary blood flow *√* Elevated systemic and pulmonary vascular resistance *√* Impaired ventricular function *√* Decreased venous return, cardiac compliance, and cardiac output. *√* Decreased contractility	*√* Acute myocardial infarction (AMI) *√* Arrhythmias *√* Cardiomyopathies: dilated, restrictive, hypertrophic *√* Cardiovascular collapse
Arrest related lung injury	*√* Reperfusion injury *√* Endothelial damage *√* Systemic inflammation *√* Activation of clotting cascades *√* Ventilation-perfusion mismatch (ventilatory pressure) *√* Basal atelectasis	*√* Pneumonia *√* Acute respiratory distress syndrome *√* Pulmonary thromboembolism
Arrest related liver and kidney injury	*√* Reperfusion injury *√* Reduced blood flow *√* Reduced urine output	*√* Hypoxic hepatitis/acute liver failure *√* Acute kidney injury

During resuscitation, the relationship between tissue oxygenation, energy demand, and cellular function is complex and strictly related to intracellular pH.

**Fig. 5:**
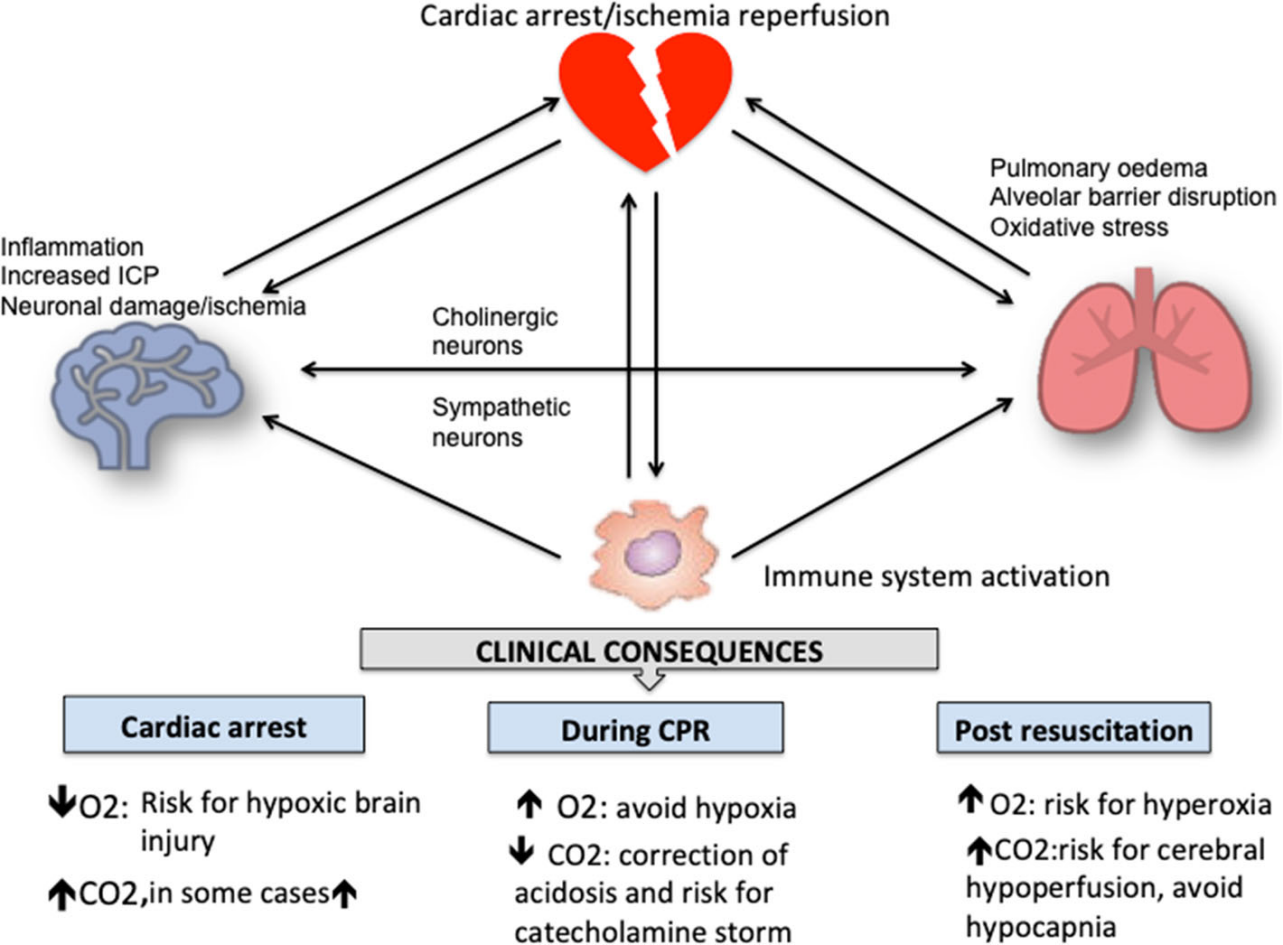
Systemic effect of cardiac arrest and clinical implications in the ventilator targets to achieve. Catecholamine release, with impaired mitochondrial metabolism, can activate immunology response. Neuronal and immune responses are rapidly activated during cardiac arrest and trigger a pathway of systemic neuroendocrine and immune responses which have important systemic consequences. Global cerebral ischemia and immunological disturbances induce microgliosis, damage of blood-brain barrier, and cerebrovascular system. Endothelial dysfunction and blood brain barrier permeability are crucial in above pathological processes. The brain-lung or brain-heart-lung couplings and their disturbances may be considered as modulator of post ischemic injury, peripheral inflammation, and multiorgan dysfunction, observed in post cardiac arrest patients. Post ischemic induction of necrosis, apoptosis, and systemic inflammation predisposes to neuronal damage and poor recovery. Pulmonary complications following cardiac arrest, through cerebral ischemia or immunological activation, can result in neurogenic pulmonary edema, ARDS, or pneumonia. The clinical consequences of these pathophysiological pathways are different according to three different phases: cardiac arrest, cardiopulmonary resuscitation, and post resuscitation. ARDS, acute respiratory distress syndrome

Evidences regarding the levels and changes in oxygen and carbon dioxide during and after cardiac arrest as well as of optimal carbon dioxide and oxygenation targets are scarce, but the above discussed pathophysiological changes during cardiac arrest may help to guide clinical practice (Fig. 5).

During cardiac arrest, oxygen and carbon dioxide levels depend on the cause of pre arrest (respiratory or cardiological, hypothermia, etc.); two recent studies showed that during cardiopulmonary resuscitation, patients presenting with hypoxia had lower survival rates than those with normoxia or hyperoxia; in these studies, hypercapnia and respiratory acidosis were common, but no association between the severity of acidosis or hypercapnia with outcome was found [51, 52]. These results could be related to a quick decrease of carbon dioxide and pH normalization that could have harmful effects [53, 54].

Other authors showed that hyperventilation and hypocapnia during cardiopulmonary resuscitation can worsen outcome by reducing cardiac output and pulmonary blood flow [43, 44].

Therefore, current recommendations suggest to avoid hypoxia during cardiac arrest patients by using high FiO_2_ 100% and a tidal volume of 500 ml, and respiratory rate of 12, minimizing acute changes on PaCO_2_ [55] (Fig. 5).

In the post resuscitation phase, attention should be paid on FiO_2_ administration, which should be titrated in order to avoid both hypoxia and hyperoxia. The use of high FiO_2_ = 100% can cause extreme hyperoxia and can result in higher release of neuron specific enolase, a biomarker of brain injury, when compared to lower FiO_2_ [46, 47]. Peng et al. [20] in an experimental ischemia reperfusion injury model showed that hyperoxia after ischemia/reperfusion injury may be predictor of elevated oxidative stress, myocardial destruction, ventricular injury, and inflammation, compared to normoxia.

Ebner et al. [49] showed that both hyperoxemia or hypoxemia exposure after out hospital cardiac arrest are not associated with poor neurological outcome at 6 months, and that highest serum levels of neuronal injury marker -Tau (s-Tau) are not significantly correlated with PaO_2_ values after 48 or 72 h from ROSC.

Hypocapnia should be avoided in order to optimize cerebral perfusion and mitigate ischemia-reperfusion injury due to post cardiac arrest oxidative stress, but the correction of hypocapnia should take in consideration the pH, catecholamine administration, and intracellular pH. In this phase, also the correction of hypercapnia should be performed with caution; some authors even suggest a correlation between good outcome and moderate hypercapnia by a potential effect of attenuation in the above mentioned excitotoxicity processes [50]. However, a recent study showed that hypercapnia, especially when associated with acidosis, is associated with poor outcome, and the current recommendation is to aim for normoventilation with a PaO_2_ of 4.5–6.0 kPa [31, 51](Fig. 4).

Patients after cardiac arrest also present with a variety of pulmonary complications, related to lung injury, aspiration, pulmonary ischemia-reperfusion injury, ventilator induced lung injury, and systemic inflammation and infections, which can affect oxygenation and ventilation.

Post cardiac arrest patients are at high risk for acute respiratory distress syndrome (ARDS), but the frequency of ARDS in this population is not well characterized. Two hypotheses have been proposed to explain the development of ARDS in these patients: the first theory includes the indirect activation and proliferation of neutrophils with progressive inflammatory alveolar infiltrates by activation of the immune system after cardiac arrest. Another hypothesis focuses on direct epithelial barrier damage related to ischemia reperfusion injury and endothelial cells activation and dysfunction [52]. The consequent overexpression of adhesion molecules, secretion of cytokines and chemokines, activates the sequestration of neutrophils, which transmigrate across the endothelium and epithelium into the alveolar space and releases cytotoxic and proinflammatory compounds [52–55].

Gas exchange impairment after cardiac arrest can be also related to myocardial dysfunction and consequent pulmonary edema, as well as impairment of the immune system with increased predisposal to infections, with frequency up to 70% [56]. Finally, therapeutic hypothermia may increase the risk of infections [57]. In fact, post resuscitation syndrome presents characteristics of “sepsis like syndrome,” with high levels of cytokines and endotoxin in plasma which are similar to patients with sepsis and predispose to infections and longer mechanical ventilation [58] (Table 1).

Altogether, attention should be paid in the optimization of mechanical ventilation, in particular in the difference phases of cardiac arrest, during and in the post resuscitation period (Fig. 5), aiming to avoid hypo- and hyperoxia as well as maintain carbon dioxide levels and pH within physiological ranges.

## Conclusions

The pathophysiological effects of cardiac arrest on systemic homeostasis are complex. The appropriate ventilator strategies are not fully understood, and oxygen and carbon dioxide targets should take in consideration several pathophysiological factors including the intracellular and extracellular pH as well as the use of catecholamines and hypothermia. In general, hypoxia (< 60 mmHg) and hyperoxia (> 300 mmHg) should be avoided as well as hypocapnia (< 30 mmHg) and hypercapnia (> 50 mmHg). Large prospective randomized controlled studies are warranted to further explore the pathophysiological basis of this theory and define the optimal setting of mechanical ventilation in patients after cardiac arrest.

## Data Availability

Not applicable

## Abbreviations

ARDS: Acute respiratory distress syndrome
ATP: Adenosine triphosphate
COX-2: Cyclooxygenase-2
DAMPS: Damage-associated molecular patterns
IL 1: Interleukin-1
iNOS: Nitric oxide synthase
MLCK: Myosin light-chain kinase
MODS: Multiple organ dysfunction syndrome
NO: Nitric oxide
ROS: Reactive oxygen species
ROSC: Return of spontaneous circulation
TLR 4: Toll like receptor
TNF: Tumor necrosis factor
VILI: Ventilator-induced lung injury
